# A Study of Morphological Characteristics of Lung Fissures and Trachea in the Indian Population

**DOI:** 10.7759/cureus.22568

**Published:** 2022-02-24

**Authors:** Arun H Narasannaiah, Ali Z Anwar, Amit Pandey, Doddabasappa Belagavi, Syed Althaf, S V Reddy, Raghavendra Harsha, Siddharth Jain, Mohammed A Ali, Pratham Batra

**Affiliations:** 1 Surgical Oncology, Kidwai Memorial Institute of Oncology, Bangalore, IND; 2 Medical Oncology, Kidwai Memorial Institute of Oncology, Bangalore, IND; 3 Forensic Medicine, Bangalore Medical College and Research Institute, Bangalore, IND

**Keywords:** cadaver, anatomy, lung morphology, body height, tracheal length

## Abstract

Tracheal length and lung anatomy have been rarely studied; however, the anatomy of the lung has been shown to vary significantly. Moreover, the surgery regarding trachea are few, and hence the surgeons do not have extensive experience in the trachea.

Objective: We aimed to study the variations of the lung anatomy and the relation between tracheal length and body height in the Indian population.

Materials and methods: This is an observational study to observe the tracheal length in relation to body height and sex and gross morphological anatomy of the lung in 70 cadavers. The data was collected from the forensic department of Bangalore Medical College and Research Institute (BMCRI), and further analysis was done at Kidwai Memorial Institute of Oncology.

Results: Deviation from normal lung morphology was seen in 37.86% of the specimens studied. The tracheal length (average, 9.97 cm) correlated with the body length (average, 147.02 cm) with a Pearson coefficient of 0.806 (p value=0.001)

Conclusion: The study of lung fissure morphology guides clinicians in understanding and planning lung disease treatment, especially lobectomy/segmentectomy surgeries. The information of the average length of the trachea with respect to body height in a given ethnicity will help during endotracheal intubation and tracheal surgical planning.

## Introduction

There have been very few studies on the anatomy of the lung and trachea length. The right lung is by its two fissures, one horizontal and one oblique, dividing it into three lobes: upper, middle, and lower lobes, Similarly the left lung is divided into two lobes (upper and lower) along a horizontal fissure [1]. However, numerous studies have shown that the anatomy of the lung varies significantly between individuals; for example, a study by Craig and Walker shows that this variance takes the form of atypical fissures [2].

Tracheal length varies from 10 cm to 12 cm. The trachea comprises 12-16 cartilages and is correlated with age, sex, and ethnicity [3]. Furthermore, trachea surgeries are performed infrequently, and consequently, surgeons do not have extensive experience with the trachea. The length of the trachea that can be safely resected by a surgeon and the length of the endotracheal tube needed by an anesthesiologist is generally based on the experience of senior medical professionals.

Similarly, accurate anatomical knowledge of the lungs is needed to interpret radiological findings and is crucial during surgery. In many diseases, segment localization is needed for proper resection, and the preoperative anatomical knowledge required to plan a lobectomy or segmental resection lowers the probability of postoperative complications such as air leaks, which lead to significant morbidity.

## Materials and methods

This is a retrospective observational study examining information on tracheal length, body length, sex, and the morphological anatomy of the lungs of 70 cadavers. The correlation between tracheal length and body length, and tracheal length and sex, were studied, as well as morphological variations in the anatomy of the lung. The local ethics committee did not request an institutional review board (IRB) since this is a retrospective study without identifying information. 

Between July 2019 and June 2020, 70 fresh cadavers were procured from the forensic medicine department of Bangalore Medical College and Research Institute (BMCRI) for the study. The inclusion criterion was age >18 years, excluding those with thoracic injuries or pathological lesions in the lungs.

During the postmortem, after routine organ retrieval from neck to pelvis for examination, we dissected out the trachea, along with the lungs, separately, to extract our data. The lungs were labeled according to the side: right or left, and the fissure anatomy was recorded, capturing the number, extent, and completeness as per the Craig-Walker classification (Table 1) [2]. 

**Table 1:** Craig and Walker classification. [2]

Grade	Description
Grade 1	Complete fissure with entirely different lobes
Grade 2	Complete visceral cleft but parenchymal fusion at the base of the fissure
Grade 3	Visceral cleft evident for part of the fissure
Grade 4	Complete fusions of the lobes with no evident fissure line

The tracheal length was measured from the inferior margin of the cricoid cartilage up to the subcarinal border of the lymph node, along with the age, sex, and height of the body. Damaged specimens were excluded from the study. All data was collated with photographs, and statistical analysis was done at Kidwai Memorial Institute of Oncology (KMIO), Bangalore, using the SPSS Statistics software version 23 (IBM Corp., Armonk, NY).

## Results

**Figure 1:**
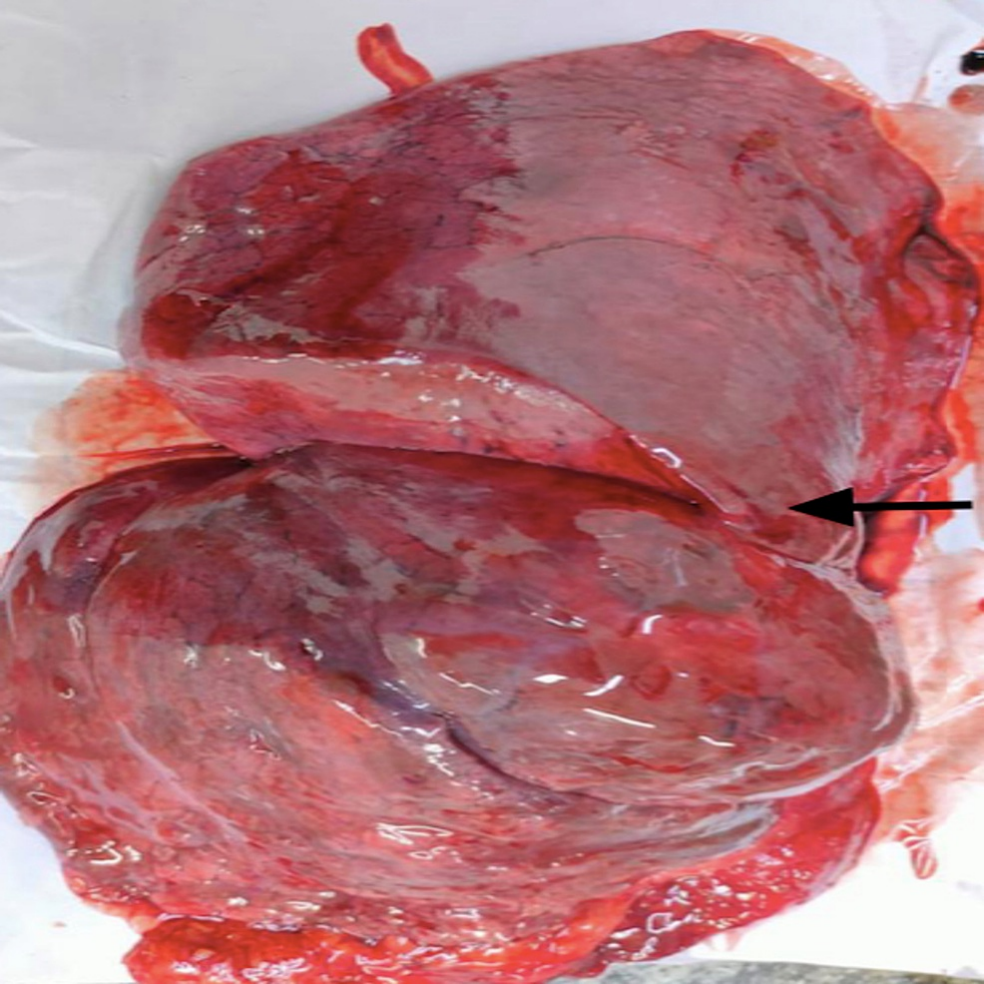
Specimen showing incomplete horizontal fissure of the right lung. The black arrow denotes the incomplete horizontal fissure in the given specimen of the right lung.

The data were collected and compiled, and various variations were discovered and are presented in subsequent tables and figures. There was an incomplete horizontal fissure, which is presented in Figure 1.

From the data collected, there were different variations in the anatomy of the right lung, although complete fissures were observed in the majority of the specimens. Variations were also observed across all specimens and are described in Table 2.

**Table 2:** Morphological characteristics of the right lung specimens.

Grade	Frequency	Percent (%)
Complete fissure (I)	44	62.9
Complete cleft but parenchymal fusion at the base (II)	5	7.1
Visceral cleft evident for part of the fissure (III)	21	30.0
Complete fusion (IV)	0	0
Total	70	100

**Figure 2:**
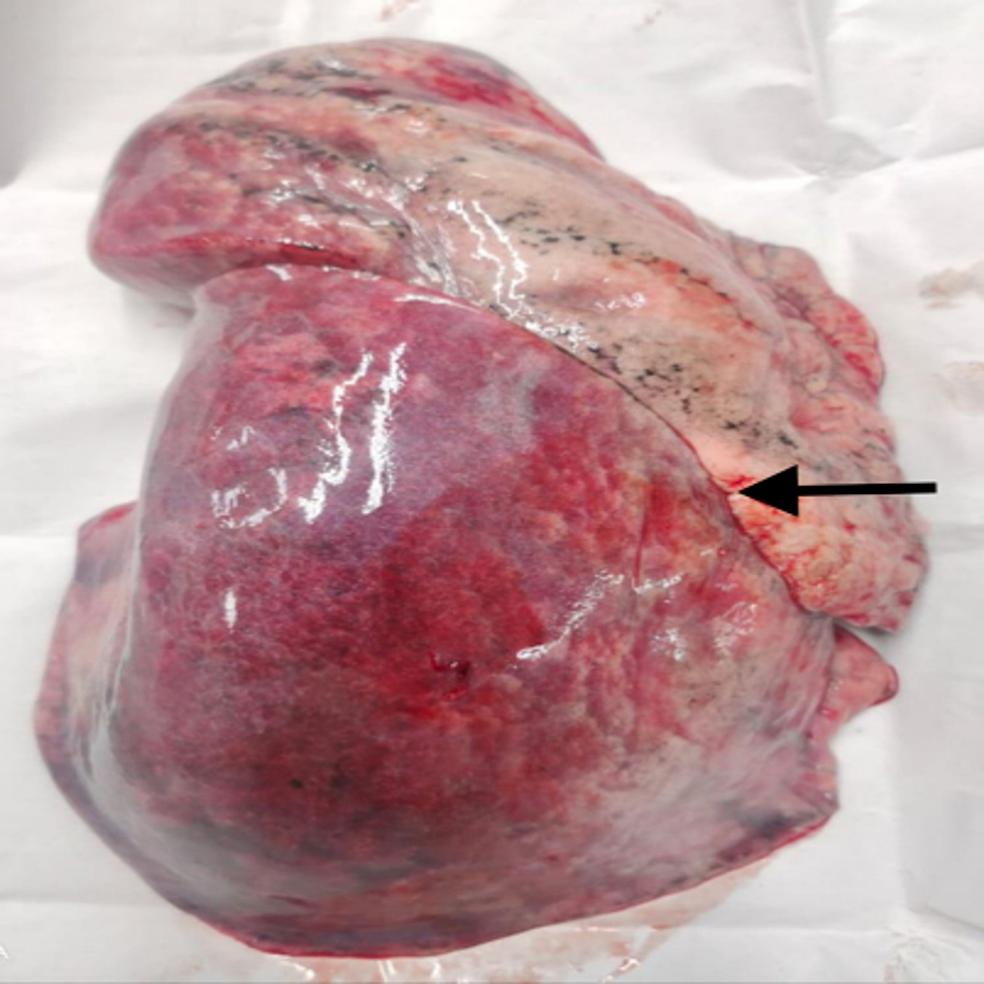
Specimen showing oblique fissure in the left lung. The black arrow denotes the complete oblique fissure in the given specimen of the left lung.

There was a complete fissure in the majority of the left lung specimens collected (Figure 2). A single specimen had a complete fusion of the fissure, and there were various other variations (Table 3).

**Table 3:** Morphological characteristics of left lung specimens.

Grade	Frequency	Percent (%)
Complete fissure (I)	43	61.4
Complete cleft but parenchymal fusion at the base (II)	3	4.3
Visceral cleft evident for part of the fissure (III)	23	32.9
Complete fusion (IV)	1	1.4
Total	70	100

**Figure 3:**
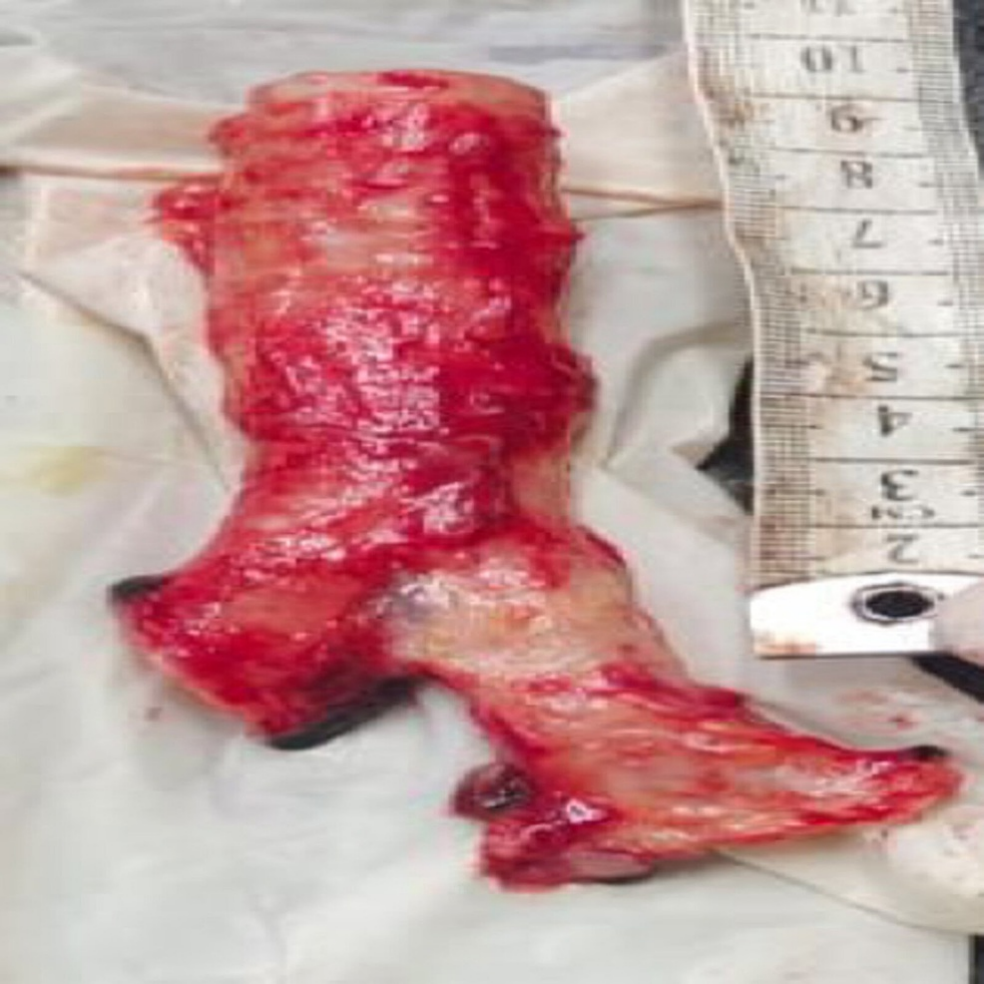
Trachea specimen with length measurement.

The average tracheal length, measured from the lower border of the cricoid cartilage to the subcarinal area, was 9.97 cm (Figure 3), and the average height of the individual was 147.02 cm. The study sample comprised 52 male and 18 female cadavers. For the males, the average tracheal length was 10.27 cm (+/− 1.52 cm), and the average height was 150.50 cm (+/− 15.28 cm). Among the females, the average tracheal length was 9.00 cm (+/− 1.30 cm), and the average height was 137.00 cm (+/− 18.77 cm). There was a positive correlation between tracheal length and body height. The overall Pearson's coefficient was 0.806 (p value = 0.001). Pearson's coefficient was 0.776 (p value = 0.001) for males and 0.792 (p value = 0.001) for females (Table 4). 

**Table 4:** Relation between body length and tracheal length.

Number	Body length	Tracheal length	Pearson's coefficient
Total: 70	147.02 (+/- 17.17) cm	9.97 (+/- 1.56) cm	0.806 (p value = 0.001)
Male: 52	150.50 (+/- 15.28) cm	10.27 (+/- 1.52)cm	0.776 (p value = 0.001)
Female: 18	137.00 (+/- 18.77) cm	9.00 (+/- 1.30) cm	0.792 (p value = 0.001)

## Discussion

Development of lungs occurs during the 6th week of embryo development, and the architecture of the bronchopulmonary segments is completed by the 14th week. The spaces between the bronchopulmonary segments then close, and wherever they do not close, the spaces develop into fissures. Partial obliteration or non-obliteration can result in the formation of incomplete or absent fissures. An accessory fissure results from the non-obliteration of spaces that would normally be obliterated. The development of the trachea ensues with the formation of the longitudinal tracheoesophageal septum, which divides the combined tracheal groove and leads to the differentiation of the foregut into trachea and esophagus during the 4th week of development. The left and right main bronchi form during the 5th week [4].

In separate studies, tracheal length has been measured using methods such as chest X-ray, computed tomography, flexible bronchoscopy, and cadaver specimens. These studies have also proposed that tracheal length may be related to the height of an individual [5]. In study the trachea was observed to be 11 cm in cadavers and slightly longer in living adults [6]. In a study conducted by Kamel et al., the tracheal length was observed to be 8-12 cm in vivo but was 2 mm shorter in cadavers [7]. A comparison of the findings of different studies on tracheal length and body length is presented in Table 5.

**Table 5:** Comparison between different studies for the association of tracheal length and body length.

Study	Association between body length and tracheal length	Body length (cm)		Tracheal length (cm)	
		Male	Female	Male	Female
Munguia et al. [8]	None	169 (+/- 6)	161 (+/- 7)	9.1 (+/- 0.9)	8.6 (+/- 0.5)
Cinar et al. [9]	Present	168 (+/- 5.6)	160 (+/- 6.4)	8.7 (+/- 1.1)	8.5 (+/- 0.6)
Pang et al. [10]	None	179 (+/- 8)	163 (+/- 8)	13.6 (+/- 1.4)	11.8 (+/- 1.3)
This study	Present	150 (+/- 15.28)	137 (+/- 18.77)	10.27 (+/- 1.52)	9.00 (+/- 1.30)

In this study, we used the Craig-Walker classification [2]. Grade 1 fissures were present in the right lungs of 44% of the cadavers, grade 2 fissures in 5%, and grade 3 fissures in 21%. In the left lungs, grade 1 fissures were observed in 43% of the cadavers, grade 2 fissures in 3%, grade 3 fissures in 23%, and grade 4 fissures in 1%. In a study conducted by Thapa et al. [11], incomplete oblique fissures were observed in 30% of the cases, an incomplete horizontal fissure was observed in 50% of the lungs, and a normal lung architecture was observed in 85% of the cases. In a study conducted by Meenakshi et al. [12], 36.66% of the right lungs and 46.66% of the left lungs had incomplete oblique fissures, and accessory fissures were observed in 10% of the left lungs and 3.3% of the right lungs examined. In a study by Lakshmi et al. [13], 25 lungs were examined, and only one lung had an absent horizontal fissure, while eight had an incomplete horizontal fissure.

This study has limitations; it is retrospective in nature and uses case records. The study was based on cadavers, and there may have been pathological or physiological incidences that eluded us, which may have influenced the morphology of the lungs during the lifetime of the individual. Lastly, the study sample was obtained from a tertiary hospital located in South India, and consequently, the results and conclusions derived may be applicable only to individuals of Indian ethnicity.

## Conclusions

Lungs have varied anatomy, and knowing the degree of incompleteness of the fissures through an accurate description of the morphology of the lungs can facilitate a deeper understanding of the development of lesions such as pneumonia lesions and pleural effusion and the spread of diseases contracted through the lungs. The study of lung fissure morphology guides clinicians in understanding lung diseases and planning treatment, especially lobectomy and segmentectomy surgeries. The average length of the trachea in relation to body height among a given ethnic group is thus useful during endotracheal intubation and planning tracheal surgeries.
